# Fake news, misinformation, disinformation and supply chain risks and disruptions: risk management and resilience using blockchain

**DOI:** 10.1007/s10479-023-05242-4

**Published:** 2023-03-08

**Authors:** Pythagoras N. Petratos, Alessio Faccia

**Affiliations:** 1 Coventry University Business School, Coventry, UK; 2 Birmingham University Business School, Dubai, UAE

**Keywords:** SCRM, Fake news, Misinformation, Disinformation, Resilience

## Abstract

Fake news, misinformation and disinformation have significantly increased over the past years, and they have a profound effect on societies and supply chains. This paper examines the relationship of information risks with supply chain disruptions and proposes blockchain applications and strategies to mitigate and manage them. We critically review the literature of SCRM and SCRES and find that information flows and risks are relatively attracting less attention. We contribute by suggesting that information integrates other flows, processes and operations, and it is an overarching theme that is essential in every part of the supply chain. Based on related studies we create a theoretical framework that incorporates fake news, misinformation and disinformation. To our knowledge, this is a first attempt to combine types of misleading information and SCRM/SCRES. We find that fake news, misinformation and disinformation can be amplified and cause larger supply chain disruptions, especially when they are exogenous and intentional. Finally, we present both theoretical and practical applications of blockchain technology to supply chain and find support that blockchain can actually advance risk management and resilience of supply chains. Cooperation and information sharing are effective strategies.

## Introduction

Fake news, misinformation and disinformation have significantly increased over the past years, profoundly affecting societies and supply chains. This paper examines the relationship and risks of these forms of information with supply chain disruptions and proposes blockchain applications and strategies to mitigate and manage such risks. While fake news is not a new phenomenon, a major proliferation of fake news occurred in the 2016 US Presidential elections, and they attracted much attention and became a public concern (Grinberg et al., 2019). The concern has not only been because of the significance of the well-functioning of democracy and the importance of the US Presidential election, not only for the US but also for the rest of the world, but also due to the vast size of fake news and new forms of media communications, namely social media.

Social media enabled new operations in communications, like easy access, quick spread, targeted audiences, and lack of controls. For example, Allcott and Gentzkow (2017) found that Facebook’s fake news during the 2016 elections was shared around 38 million times, translating to approximately 760 million clicks. During the 2016 Presidential election, fake news on Twitter accounted for nearly 6% of all news consumption, but it was heavily concentrated on some users (Grinberg et al., 2019). Finally, the production of fake news, or disinformation, as we will discuss later, came in many cases from foreign countries and actors (NASEM, 2018). Fakes news, misinformation, and disinformation remain significant risks to elections worldwide.

Another surge of fake news, misinformation and disinformation appeared during the COVID-19 crisis, known as the infodemic. “An infodemic is too much information, including false or misleading information in digital and physical environments during a disease outbreak […] It causes confusion and risk-taking behaviours […] An infodemic can intensify or lengthen outbreaks […] With growing digitisation – expanding social media and internet use – information can spread more rapidly. It can help fill information voids more quickly and amplify harmful messages.” (WHO, 2022). Thus, the infodemic has a significant effect on strengthening and extending the COVID-19 pandemic. The COVID-19 pandemic had substantial negative effects on supply chains, and there are changes and digitisation in supply chains, while risk management resilience is a top priority (EY, 2021). “*Entering the COVID-19 pandemic wreaked havoc on supply chains*”, and there are after-shock risks still disrupting them (Ivanov, 2021). Supply chain disruptions during COVID-19 have been largely idiosyncratic, impacting different firms at different times for different reasons (Dunn, 2021). COVID-19 differs from other supply chain disruptions, such as the financial system and demand and supply (Moritz, 2022). Therefore, the risks and impact on supply chains of COVID-19 are different from previous literature, and their study contributes to the literature.

Scholars and practitioners emphasise that managing disruption and risk in the supply chain should be a crucial capability for firms (Shekarian & Mellat Parast, 2020). Nevertheless, despite the criticality of supply chains, businesses and economies face unprecedented risks and disruptions. Companies impacted by supply chain disruptions have experienced ‘adverse consequences and dramatic financial losses’ (Bugert & Lasch, 2018). In order to better manage supply chain risks and ensure profitability and continuity (Tang, 2006), organisations have used Supply Chain Risk Management (SCRM). SCRM is a popular research area, and interest in this subject has grown (Ceryno et al., 2013). Numerous literature reviews have confirmed the importance and extent of SCRM over the years (Tang, 2006; Khan et al., 2008; Manuj & Mentzer, 2008; Rao & Goldsby, 2009; Tang & Nurmaya Musa, 2011; Colicchia & Strozzi, 2012; Sodhi et al., 2011; Ceryno et al., 2013; Fahimnia et al. 2015; Ho et al., 2015; Snyder et al., 2015; Rajagopal et al., 2017; Prakash et al., 2017; Bugert & Lasch, 2018; Elock Son, 2018; Seipp et al., 2020; Shekarian & Mellat Parast, 2020).

Tang (2006), based on the definitions of SCRM, makes a distinction between two dimensions (i) Supply chain risks and disruptions; and (ii) the Mitigation Approach concerning supply management, demand management and, most importantly, in our context, information management. The second dimension overlaps with another important field of study, Supply Chain Resilience (SCRES). SCRES “reduces the impact of disruptions by identifying strategies that allow a supply chain to react to a disruption while recovering to its original functional state or better” and has received more attention in recent years (Shekarian & Mellat Parast, 2020). While not as extensive as SCRM, there are several reviews on the literature (Ali et al., 2017; Durach et al., 2015; Hohenstein et al., 2015; Katsaliaki et al., 2021; Kochan & Nowicki, 2018; Tukamuhabwa et al., 2015) displays its substantial size and growing importance.

### Motivation

**Fig. 1:**
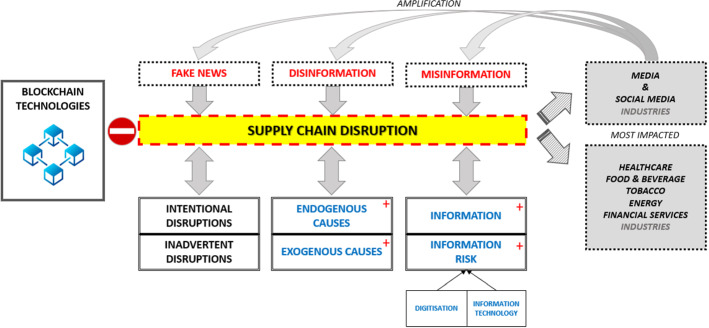
DuHadway et al. (2019) Framework enriched

This paper builds upon the widely used literature on SCRM and SCRES. The main purpose is to assess information risks in fake news, misinformation and disinformation on supply chain disruptions. In addition, DuHadway et al. (2019) found that “*strategies to mitigate supply chain risk tend to treat disruptive events as homogenous, despite having different causes and requiring different risk management strategies*”, and they developed a framework to understand risk management strategies the source of the disruption as endogenous or exogenous to the supply chain if the cause was an intentional or inadvertent act. Based on DuHadway et al. (2019), we expand and enrich their framework about fake news, misinformation and disinformation. As we will discuss later, this misleading or wrong information presents both exogenous and endogenous causes and intentional and inadvertent effects (see Fig. 1).

Tang (2006) and Tang and Nurmaya Musa (2011) emphasise the importance of information management in supply chains. They combine definitions of supply chain management. In the context of SCRM, they define it as the management of information flows. After reviewing the literature, they conclude that information flow risk has received relatively less attention (Tang, 2006; Tang & Nurmaya Musa, 2011). Motivated by this limited attention to the information element, we argue that information and information risks are critical in supply chain disruptions, especially as they become even more important as digitisation and Information Technology (IT) are gaining pace (see Fig. 1).

Fisher Ke et al. (2015) find support that industry characteristics have an impact on global supply chains. It is hard to evaluate the impact and disruptions of misinformation, disinformation and fake news on specific countries, sectors/industries and supply chains. Nevertheless, this paper contributes by initiating an attempt to study how supply chains were affected, by the quality of information, especially during COVID-19 and the related infodemic. Industries that are foremost impacted by misleading information are media and social media. Media and Social media are crucial since they can distort and amplify misleading information. For example, Tasnim et al. (2020) examined the impact of Misinformation on COVID-19 and found that it promoted erroneous practices and disrupted health and food supply chains. There is exhaustive literature on how misleading information and COVID-19 substantially impacted supply lines in healthcare. In addition, there are effects and disruption caused by fake news and disinformation on other industries such as tobacco, energy, food and the financial industries (Akhtar et al., 2022).

Blockchain is an emerging information technology that can provide several potential applications related to supply chains (Helo & Hao, 2019). In times of increased risk and uncertainty, Blockchain can manage and improve supply chain resilience (Etemadi et al., 2021). To contribute to the limited literature on information risks of SCRM (Supply Chain Risk Management) and the great potential of blockchain applications in SCRES, we identify two major research questions.

- RQ1 What are the information risks, particularly concerning fake news, misinformation and disinformation, and how can they disrupt supply chains?
- RQ2 How do blockchain technologies improve information management risk management and increase supply chain resilience?

In answering these research questions, we use the SCRM literature to examine information risks and how they affect supply chains. Then by utilising SCRES, risk management and mitigation strategies and practical applications based on Blockchain are recommended. This study’s main contribution is expanding on the limited literature on information management and risks in the supply chain. Most importantly, it provides an innovative perspective by analysing fake news, misinformation, and disinformation concerning information flows and risks. This part is a new contribution, as far as we are aware. The second major contribution is recommending risk management and mitigation strategies and applications of emerging blockchain technologies. While there is some recent literature on blockchain and supply chains, we expand on it and focus on the information aspects.

The paper is organised as follows.

- Section 2 reviews the most relevant literature on SCRM and SRES, mainly focusing on information management. We also review basic literature on Blockchain about supply chains and associated risks.
- Section 3 is devoted to the analysis of information, in particular fake news, misinformation and disinformation.
- Section 4 describes and relates information processing theory to supply chains and disruption.
- Section 5 provides propositions and strategies for information risk management. We examine various blockchain applications in supply chains and recommend risk management and mitigation strategies to reduce disruption and improve resiliency.
- Finally, in the last Section 6, we conclude the paper by summarising the main findings and contributions, limitations and outline future research.

## Literature review

Below, we review four primary bodies of literature to theoretically and practically analyse the impact and risks of misleading information on supply chain disruptions and how blockchain technology can help manage such risks, following a similar methodology to the literature review by DuHadway et al. (2019). We first review the role of information in SCRM. Based on Sodhi and Son (2012), we look at different aspects of SCRM, split into subsections to better understand diversity. Then we describe Blockchain and examine how it can have numerous advantages for the supply chain sector. We define misleading information, particularly Fake News, Misinformation and Disinformation. Consequently, after examining the role of information in SCRM and the nature of misleading information, we review organisational information processing theory (OIPT), which constitutes the main framework of our analysis in the next section.

### The importance of information

Information is an overarching theme in supply chain management. While information management and risk analysis present some literature limitations, the use of information has wide applicability. Prakash et al. (2017) emphasise the central role of information in Supply Chains Management “a supply chain (SC) has to manage the flow of a large amount of information and a variety of products across all its stage”. Information is essential to decision-makers in supply chain management. Rajagoral et al. (2017), in a review of decision-making models for supply chain risk, find that information risks are the least addressed and receive limited, and we argue disproportionate attention. Rao and Goldsby (2009) argue that decision-maker-related risks include decision-maker knowledge/information and information-seeking behaviour. Lavastre et al. (2012) suggest that effective SCRM is based on collaboration and relevant information exchanges and discuss the benefits of a comprehensive information system for the entire supply chain to anticipate all possible contingencies. Without appropriate information, it is impossible to anticipate risks and contingencies, and decision-makers cannot effectively react.

The literature captures some information aspects related to fake news, misinformation and disinformation, such as quality and visibility. Katsaliaki et al. (2021) conclude that quality information sharing and investing in appropriate technologies, including Blockchain, helps with visibility in the supply chain and enhance trust and cooperation to better protect against disruptive events. Visibility is crucial to monitor and managing information quality and identifying fake news, information, and disinformation. Baryannis et al. (2019) discuss the problem of interpretability and how practitioners can understand information to make decisions that can mitigate or prevent risks. Interpretability is valuable since the interpretation of information can distinguish it from misleading or wrong information. Another interesting conceptualisation of risks is relational risks (Manuj & Mentzer, 2008), such as distorting information (Baird & Thomas, 1985).

#### Information flow risks and information distortion

The major part of the related literature concerns information flows and risks. Spekman and Davis (2004) studied information flows early as six risk sources. They examined the extended enterprise and argued that achieving transparency and visibility of information throughout the supply chain and utilising technology can bring significant benefits and competitive gains (Spekman & Davis, 2004). In addition, they analyse different aspects of information, notably visibility and sharing of information, topics repeated in the literature, and we examine them in more detail just below. Jüttner et al. (2003) used the perspective that supply chain disruptions affect the flow of information, and therefore supply chain risks comprise any risks for the information. There can be disruptions and ‘chaos effects’ in supply chains from factors such as distorted information throughout the supply chain or simply a lack of understanding amongst organisations (Jüttner et al., 2003).

It is useful for our study to briefly examine the concept of information distortion, which can largely overlap with fake news, misinformation, and disinformation. There is a growing literature attempting to understand information distortion better and flows in the supply chain (Sahin & Robinson, 2002), but it seems that this literature has not significantly grown during the last couple of decades. The literature can be separated into two phases, an early one focusing on understanding complexities and the information distortions, including the “bullwhip effect”, and a second phase targeting information coordination and sharing. The latter phase’s themes remain the focus of the more recent literature. Lee et al. (1997) connect information distortion with the bullwhip effect by studying a specific aspect of demand information flow. Holweg and Bicheno (2016) describe different sources of distortions, and they can be directly or indirectly related to information flows, including the ‘Forrester’ (bullwhip) and the ‘Burbidge’ effects.

Forrester’s (1961) seminal work is important because of the bullwhip effect and because he recognises the protagonist’s role of information by suggesting that an information network integrates other flows. In simple terms, Forrester (1961) acknowledged the challenges of information and that not perfect or inaccurate information increases variability and, thus, risk as we move upwards in the supply chain. He also linked decision-making with varying flows of information that are converted into control signals for other flows: “Information is the input to a decision, and the decisions are affected by all influences that act on the information flows. Information can be distorted in other ways than by delays and amplification. Information is modified […] information is interpreted differently by different people and organisations. Prejudices, history, integrity, hope and the internal political environment of an organisation all bias information flows. The information contains errors, random noise, and unknown perturbations from external sources.” (Forrester, 1961). The interpretation of information and especially perturbations by external sources might coincide with fake news, misinformation, and disinformation. In this context, a major advantage of this research lies in the distortion and amplification mechanisms.

#### Information, demand and supply risks

Despite the importance of information, especially in early literature, information risks are least addressed, needing more attention (Rajagopal et al., 2017). As noticed above, information risks are often part of demand risk. Shekarian and Parast’s (2020) review of the literature argues that there is no consensus and definition for each type of supply chain risk. Information risks are incorporated in demand risks as potential disturbances in the flow of information, insufficient or distorted information, and supply risks as potential disturbances within the network. While it is recognised that there are risks in information flows and used to keep all supply chain elements updated, examples are often connected to demand and supply, such as order, delivery, and inventory status (Tang & Nurmaya Musa, 2011).

#### Collaboration, information sharing and information systems

Tang (2006) emphasises the importance of information management as the main approach for managing supply chain risks and classifies the literature on managing information risks in supply chain visibility and information sharing. There is extensive literature on information sharing. To understand the significance of collaboration, SCRM is defined as ‘the management of risks through coordination and collaboration’ (Tang, 2006). Different mechanisms exist to coordinate/collaborate; one way is to access different information available to supply chain partners (Tang, 2006). In another survey of SCRM, Colicchia and Strozzi (2012) argue that the focus goes beyond the single company and involves the collaborative sharing of information and best practices of supply chain partners. Finally, an empirical review finds that effective SCRM is based on collaboration with timely and relevant information exchanges (Lavastre et al., 2012). The question that arises, and it is key for examining fake news, misinformation and disinformation on supply chain disruption, is “what is the quality of the information that partners share?” Some of this information might be wrong or misleading, intentionally or unintentionally.

Within this broad coordination and collaboration area, specific disruption and risk information can be shared among partners. Most papers on supply chain disruptions assume knowledge of the disruption process exactly, but in reality, it is often difficult to estimate disruption because suppliers and partners might not be willing to share disruption information (Snyder et al., 2015). Therefore, there might be misinformation concerning supply chain disruptions in the form of a lack of inaccurate information. Nevertheless, several studies demonstrate the value of accurate disruption information (Snyder et al., 2015); therefore, examining it in more detail is useful. Risk-related information is another topic that we believe has much value in SCRM but is largely neglected. It is crucial not only because it is used to manage and mitigate risks but also because it can be hard to interpret and estimate, especially in the case of significant and unprecedented uncertainty (like COVID-19), and therefore might be subject to fake news, misinformation and disinformation. Lavastre (2012) argues that cooperation to improve supply chain visibility and share risk-related information would reduce supply chain risks.

This visibility relies heavily on good information systems and connectivity throughout the supply chain (Lavastre et al., 2012). The role of IT is, therefore, critical in communicating information throughout the supply chain, especially with the digitisation of numerous operations. Rajagopal et al. (2017) review the literature, classify operation risks, and connect information flow risks with the risks associated with information systems, security and disruption, intellectual property, information outsourcing and accuracy. Similarly, Tang and Nurmaya Musa (2011) find information flow risks from the issues of information system security and disruption, intellectual property, information accuracy and information outsourcing risk. There are internal risks, such as forecast inaccuracy and information system risks (Bugert & Rainer, 2018). IT capabilities are important for information sharing and increasing visibility, transparency and connectivity (Ali et al., 2017). Katsaliaki et al. (2021) categorise Information Systems disruptions as one of the sectors of supply chain disruption. It is needless to continue emphasising the importance of information systems in supply chain disruption. Nevertheless, information systems can be prone to misleading and wrong information and associated cybersecurity risks.

### Blockchain

The Blockchain is a secure technology that ensures transparency and is useful for storing and sharing information (Siyal et al., 2019), which records all the transactions that have taken place among users since its inception. The blockchain ledger is shared by those same users who are responsible for verifying the validity of the data sharing (Liang et al., 2017) and is currently appreciated for tracking the transfer of assets as well as for the automatic execution of smart contracts (Kongmanee et al., 2019; Szabo, 1997). Three main types of blockchains can be identified: public (open to everyone), private (where only certain users have access) and consortium (where instead of only a single organisation, multiple organisations govern the platform) (Zhang & Lin, 2018).

The mechanism is based on three principles (Bhutta et al., 2021):

- Transparency: everyone can examine the transactions registered in the Blockchain since its inception;
- Decentralisation: its network operation makes the Blockchain independent from any central control body;
- Security: the transactions made through the Blockchain are encrypted, and the data cannot be tampered with or altered.

The user must enter a key that allows registering the sharing data to carry out any transaction. This data is encrypted and grouped into “blocks”(Yeasmin & Baig, 2019) and then sent to the various nodes on the network for validation. This phase allows certifying, among other things, the parties’ identity and the transactions’ feasibility. Finally, the ledger is duplicated on the servers that make up the network (Lim et al., 2018), making it impossible to modify the Blockchain or the content of one of the blocks without the approval of all connected computers.

The Blockchain offers numerous advantages for the supply chain sector, and in particular, it allows to:

- Automate purchasing processes (Omar et al., 2021). The Blockchain allows the creation of smart contracts. When the conditions selected by the users are met, these contracts automatically execute the terms, for example, the payment of service.
- Streamline trade (Hellwig & Huchzermeier, 2019). The validation times of exchanges between suppliers and customers (contracts, signatures, orders, payments) are drastically reduced, and the management of flows and relationships with partners takes place almost in real time.
- Ensure supplies (Ahmad et al., 2021). It is possible to assign a specific tag to each product registered in the Blockchain to guarantee its supply in an extremely short time. Furthermore, information such as audit trail, storage, authenticity and certificate of ownership are all stored in the same place.
- Ensure complete traceability (Galvez et al., 2018; Shahid et al., 2020) (audit trail) and, accordingly, visibility. It provides a list of all actions carried out by users: an immutable and flawless proof that ensures the integrity of the information provided.
- Increase responsiveness (Hamida et al., 2017). The Blockchain allows for avoiding counterfeiting by identifying problems from the initial stages of the transactions (i.e., inconsistencies in the validation phase, dubious identity of a party). If there is a need to return a product, a notification is sent immediately.
- Standardise internal documents (Rieger et al., 2019). The validity of the information shared between the partners avoids the multiplication of document versions; each party involved in the exchange thus has the same available data.

However, this technology still needs to be refined by developers to be tailored for supply chain purposes (Esmaeilian et al., 2020). Other limitations concern its scalability and security (Zheng et al., 2017). It is not yet possible to process large infrastructure data—for which a millisecond return is required (Aberer et al., 2006; Esposito et al., 2021; István et al., 2018)—while the Blockchain can only process transactions at one or two digits per second (Bach et al., 2018). Therefore, not surprisingly, many multinationals, including Walmart, collaborate with IBM to develop a blockchain designed specifically for their businesses (Lacity & Van Hoek, 2021; Kamath, 2018).

Blockchain cannot solve all supply chain and logistics challenges alone. It can certainly help ensure transaction security (Singh & Singh, 2016), fraud avoidance (Hyvärinen et al., 2017) and error reduction (Nguyen & Dang, 2018). Technical developments are already planned to facilitate its application to the supply chain and associated operations. It can be argued that blockchain technology is still not very mature, especially concerning supply chains. However, the companies that started the trials underline the increase in transparency and security (Angelis & Ribeiro da Silva, 2019). Some have seen reduced costs and time to conduct operations (Pan et al., 2020). The Blockchain finds application in various fields, such as Healthcare, Transport, Finance, Food and Manufacturing (Dutta et al., 2020). Blockchain in manufacturing allows more data control, increasing the speed of retrieval, and it can help reduce the problems of information asymmetry and imperfect information. Greater data security, reliability, integrity, transparency and visibility in all production and distribution processes become essential to manage better and support Blockchain supply chains.

#### Blockchain and supply chain disruption and risks

Although the Blockchain can computerise the concept of trust, there is no lack of performance gaps, such as the ethical problem (Dierksmeier & Seele, 2019) linked to the high energy consumption required and the poor scalability of the solution, since in the face of a large number of transactions the processing time increases (Nyamtiga et al., 2019). The relationship between companies and suppliers is increasingly digital, and cyber-criminals (Wang et al., 2019) can compromise any link in the company Supply Chain in various ways to steal sensitive data or information. The most common attacks against the corporate supply chain are Watering Holes (Zimba et al., 2019) and Ransomware (Pletinckx et al., 2018): Each node involved in the supply chain is a potential risk factor, and this makes the adoption of Cyber Risk prevention and management measures essential, in addition to adequate Cyber Security tools capable of detecting third-party violations and protecting the Supply Chain from cyber threats.

#### Additional technologies in supply chain decision making

The business community has started thinking about how industry 4.0 technology could help misleading combat information, especially how it could check such misinformation and fake news (Chatterjee et al., 2022). Petratos (2021) suggest that business sectors can create and use a range of anti-misinformation, disinformation, and fake news tools. However, it is useful to strategically align such technologies with the supply-chain industry decision-making, which depends on the quantity and quality of information. While Blockchain mainly applies to information security, other industry 4.0 technologies use applications to detect and monitor misleading information. Therefore, the technologies mentioned below can provide lessons learnt and best practices and might benefit stakeholders such as operational managers, social media researchers, or practitioners.

##### 2.2.2.1 Big data analytics

Koot et al. (2021), in a systematic literature review, find that there is a fusion of the Internet of Things (IoT) and Big Data Analytics (BDA) in supply chains. Moreover, these technologies can better predict and monitor supply chains (Koot et al., 2021). In an extensive literature review, Nguyen and Dang (2018) confirm that BDA and its application in SCM have revolutionised ‘supply chains (SCs)’. They also suggest that risk management appears in a few papers and has only been exploited in detecting procurement risk (Nguyen & Dang, 2018), implying a gap in this literature. Addo-Tenkorang and Helo (2016) also review the literature on big data applications in supply chain management and suggest that as data and/or information supply-chain management data are in general confidential and sensitive, the security aspect of big data/IoT could be further investigated for authenticity, and certainly our analysis and Blockchain contributes by such investigation and solution and filling a gap in risk management.

##### 2.2.2.2 Artificial intelligence, machine and deep learning

The above literature on BDA includes several terms as Artificial Intelligence (AI) and Machine Learning (ML) (Koot et al., 2021; Nguyen & Dang, 2018; Addo-Tenkorang & Helo, 2016), indicating the fusion, complementarity and in some cases integration of technologies in SCM. Nevertheless, the literature on AI, ML and Deep Learning in SCM is distinct and extensive. Toorajipour et al. (2021) conducted a systematic literature review on artificial intelligence in supply chain management and suggested a categorisation with fields and subfields. The supply chain filed is approximately 1/3 of the literature, and there is only one paper, the subfield of SCRM (i.e. Tsang et al. 2018).

Riahi et al. (2021) perform a descriptive bibliometric analysis on the application of AI in the supply chain. They also found different industries but also classified papers on the AI algorithm or technique, and the most used technique was genetic algorithms (14 papers) which decreased both the bullwhip effect (BWE) and the cash flow bullwhip (CF-BWE), but also genetic algorithms were also used for risk assessment and present applications related to our analysis (Riahi et al., 2021). Baryannis et al. (2019) provide a comprehensive literature review of the supply chain relevant to SCRM using AI approaches and categorise existing literature according to the AI methodology used, covering topics such as Machine Learning and Big Data Analytics withing this field. It confirms once more the relationship and overlap between technologies. Most importantly, they examine and categorise the reviewed studies concerning the specific SCRM tasks and find that the vast majority of studies focus on risk response (84%), mainly supply chain models for the avoidance or mitigation of risk and uncertainty effects, while other categories deal with identification and assessment or combinations of the three categories (Baryannis et al., 2019). Some of these studies might be combined with our proposed framework and enriched with other technologies on top of Blockchain for better risk management and mitigation of types of misleading information.

Naz et al. (2022) suggest that the disruptions that occurred due to the COVID-19 pandemic created a severe need for supply chain resiliency (SCR) and conduct a systematic literature review to identify the significance of artificial intelligence (AI) for creating a resilient supply chain and solutions for supply chain risk mitigation and examine the potential contribution of AI and SCR. They find substantial evidence of significant disruptions and associated risks and propose a research framework for AI in SCR that will facilitate technological development in supply chain firms to combat sudden risks and disruptions (Naz et al., 2022). In their framework, they recommend (Proposition 2) the applicability of emerging technology like AI […] and Blockchain in creating a resilient supply chain, and our study contributes to this direction. They also emphasise the importance of good quality information sharing (Naz et al., 2022), which is a key element of this paper. It should also be mentioned that there is a growing and evolving literature on these topics, such as Nayal et al. (2021) highlighting the need to ensure reliable information and suggesting that the application of AI-ML in the context of industry 4.0 technologies like Blockchain may have a strong potential to improve information capabilities; Rodríguez-Espíndola (2020) examine the integration of AI and Blockchain with potential for augmented decision making and risk reduction, and Wamba and Queiroz (2020) that suggest that the interplay between Blockchain and artificial intelligence could contribute to creating value. This paper contributes towards this combination by providing a methodology for blockchain application and information in SCM and SCRM.

## Fake news, misinformation and disinformation

Firstly, it is essential to define fake news, misinformation, and disinformation for our study. While these terms present some commonalities and fall within the broader definition of false or wrong information, they can behave differently and have diverse effects and disruptions in supply chains. Starting from the most popular term, fake news is defined as “originally US news that conveys or incorporates false, fabricated, or deliberately misleading information, or that is characterised as or accused of doing so” (Oxford English Dictionary, 2021). It should be noted that fake news is related to media and social media. Misinformation has a broader and more generic definition that it is used by a different organisation (i.e. WHO) as “wrong or misleading information” (Oxford English Dictionary, 2021).

Disinformation, on the other hand, significantly differs from the other two, defined as “The dissemination of deliberately false information, esp. when supplied by a government or its agent to a foreign power or the media, to influence the policies or opinions of those who receive it” (Oxford English Dictionary, 2021). Disinformation is by far the most dangerous type. Since governments supply it, it can be very sophisticated, using various media and other communication channels and supported by substantial resources. Therefore, disinformation can encompass many capabilities and have considerable impact and risks. Alexander and Smith (2011) discuss a taxonomy of disinformation and define it as intentional deception, which has been applied in wars. The potential severity and application of disinformation under specific conditions are displayed in that sense.

It should be emphasised at this point that intention is a critical feature. Disinformation is, therefore, clearly intentional. In general, fake news and misinformation are considered to be inadvertent. Ireton and Posetti (2018) suggest misinformation is “not created to cause harm”. However, it could be argued that this is a grey area. Allcott and Gentzkow (2017) define fake news as “news articles that are intentionally and verifiably false and could mislead readers”, displaying some intention. The intent and intention to cause harm to differ since the latter goes one step further. Søe (2019) examine truth- to the intention/intentionality and misleadingness/non-misleadingness concerning misinformation and disinformation. After critically reviewing the literature, the balance seems to be that disinformation is intentional, while misinformation is inadvertent, concluding that disinformation ‘is intentionally misleading’ and misinformation ‘unintendedly misleading’. We adopt this distinction for our analysis.

Intent is an important term related to law motive. “In Law, [motive] This is why one committed the crime, the inducement, reason, or willful desire and purpose behind the commission of an offence. Whether the purpose was good, like helping someone commit suicide, or bad, like murder, it is not a deciding factor in guilt or innocence. However, the intent is […]It may be used by a defence attorney in punishment mitigation or by a prosecuting attorney as circumstantial evidence to prove guilt” (Black’s Law Dictionary, 2022). We do not want to expand on legal issues, but we highlighted that to display that there could be legal repercussions and therefore risks from disinformation and fake news and misinformation.

Deception is another useful concept that has been predominantly used in the military context. However, Chadwick and Stanyer (2021) define deception as an ‘identifiable actor’s prior intention to mislead results’ and propose deception as a bridging concept facilitating the study of disinformation, misinformation and misperceptions. This study is valuable because it suggests that attention must go beyond individual variables to capture media-systemic distortions in information supply (Chadwick & Stanyer, 2021). It enables our analysis since it provides an innovative feature of systemic wide information disruptions that assists in conceptualising how wrong and misleading information can disrupt supply chains. While most models discuss unique events and disruption, Chadwick and Stanyer (2021) suggest systemic-wide, continuous and persistent information issues and potential disruption.

There can be different aspects of distinguishing fake news, misinformation, disinformation and other similar concepts like deception. In the context of operations management and the purpose of this study, we are rather interested in information and not so much the different characteristics. We can use a simple dimension that can encompass other characteristics but capture the effect of disruption. Petratos (2021) argues that “quality of information” is another bridging concept, where different types of wrong and misleading information can overlap, and quality information is critical for organisations. For this study, we can argue that there can be a scale of quality of information. There can be fully positive, perfect information that can assist in the efficiency of supply chains. At the same time, fake news, misinformation, and disinformation can help dimmish the quality of information and disrupt the supply chain. Disinformation can be the worst case of information quality due to its severity, resources backing it and high level of distortion. Negative quality information can cause catastrophes and paralysis in supply chains. The quality of the information scale can be directly related to the level of disruption in the supply chain.

## Organisational information processing theory

DuHadway et al. (2017) utilise and integrate Organisational Information Processing Theory (OIPT) to explain how organisations can proactively respond and manage the risks of different types of supply chain disruption disruptions. We follow this methodology not only because the study of DuHadway et al. (2017) largely facilitates our analysis but also because OIPT is grounded on information and uncertainty (risk). Galbraith (1974) describes the main premise of the Information Processing Model as “the greater the task uncertainty, the greater the amount of information that must be processed among decision-makers”. In addition, Galbraith (1974) identifies some important dimensions; the timing of information flows to and from the decision mechanism, the frequency of decision, the capacity of the decision-maker to process information and the degree of formalisation of information flow. These dimensions can be associated with some of the concepts presented above and provide consistency to the methodology. Formalisation and processing can present some analogies regarding the easiness and ability of interpretation.

While Galbraith (1974) focused on the amount of information, later studies highlighted the quality of information. Daft and Lengel (1984, 1986) discuss the richness of communication media information and the structural characteristics of formal information systems. Stock and Tatikonda (2008) argue that the quality of information is important, but they equate it with richness (Haußmann et al., 2011). Therefore, there is also a gap in the literature on OITP concerning the quality of information, and our paper contributes to it. OITPs state that organisations are structured around information, and (information) and its management (i.e., the use of information) are the organisation’s most critical performance, while there is increasing awareness among researchers that information is perhaps the most critical organisational contingency (Fairbank et al., 2006). There is empirical support for the Information Processing theory and, most importantly, for the scope of our analysis, information processing has a positive relationship with risk management performance (Fairbank et al., 2006).

Therefore, information processing is crucial to the management and mitigation strategies for supply chain risk (DuHadway et al., 2017). Information processing theory (IPT) is a widely adopted theoretical framework to examine firms’ performance, and such studies have contributed significantly to the development of the SCRM literature (Fan et al., 2016). Although the literature emphasises that firms can effectively reduce supply chain risks and disruptions through risk information, as presented above, studies examining how firms can use the information to achieve superior performance are scant in the literature (Fan et al., 2016). Thus, our study also contributes to this lacuna in literature. We build upon the study of DuHadway et al. (2017) that connect OITP to disruption and suggest a series of propositions examining the implications of supply chain disruptions/risks. Following this framework and methodology, we similarly assess information risks from fake news, misinformation, and disinformation to supply chain disruption. Accordingly, we propose risk management and mitigation strategies based on blockchain technologies.

## Framework, propositions and strategies

**Fig. 2:**
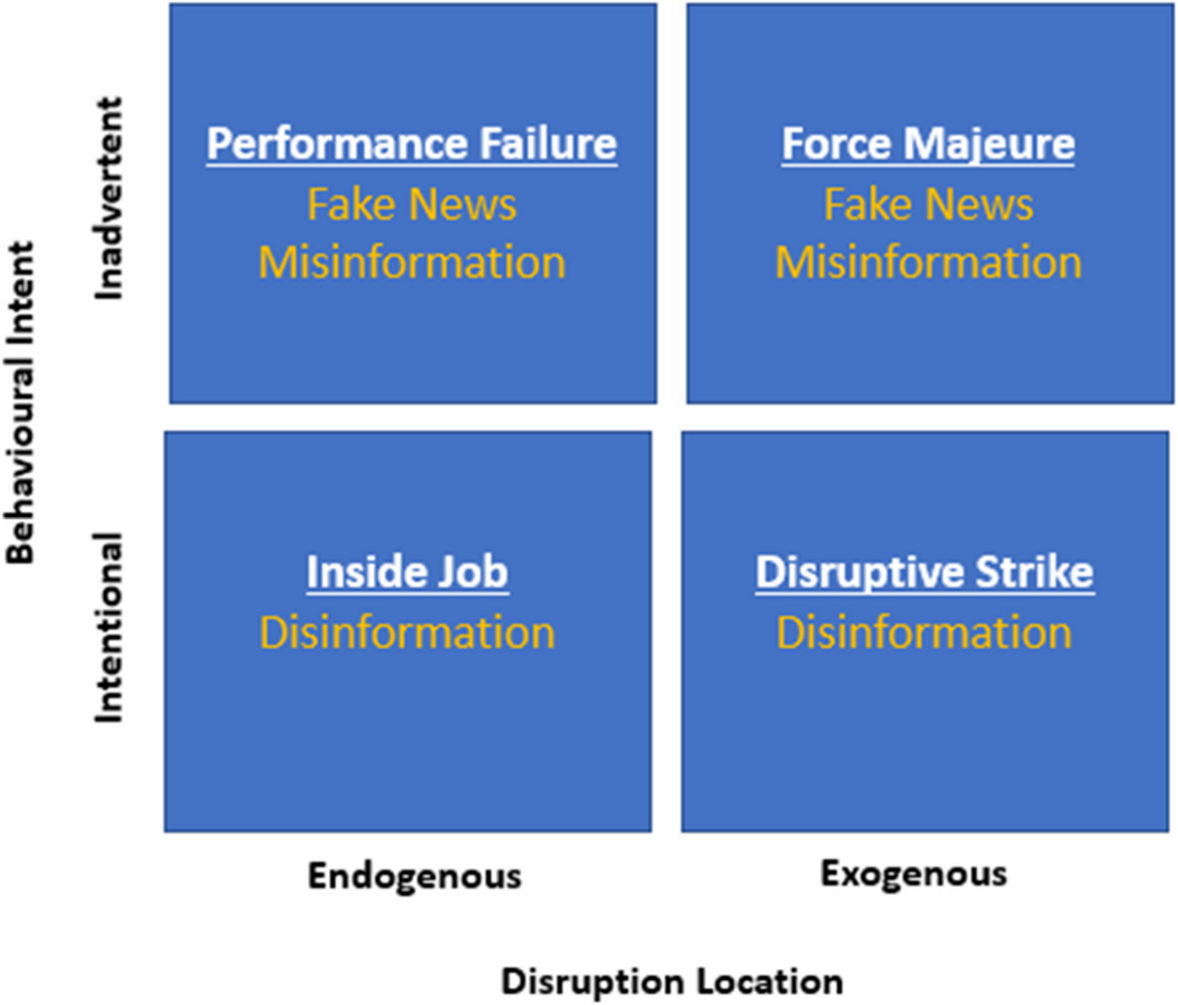
Classification of disruptive events concerning Information Types. Source: DuHadway et al. (2019)

DuHadway et al. (2019) create a framework that categorises supply chain risk/disruptions in two ways: exogenous or endogenous to the firm and inadvertent or intentional. Following this framework and methodology, we analyse fake news, misinformation and disinformation along these dimensions. When examining these information concepts above, the distinction between inadvertent and intentional was made for fake news/misinformation and disinformation, respectively. However, we still have to analyse how misleading information behaves and impacts an organisation as exogenous or endogenous. In order to do so, we construct some basic models of how low-quality information can impact and be transmitted to and within organisations and disrupt supply chains. Since there is not much literature on information and supply chain disruption, we use alternative literature. OITP facilitates this analysis and, in particular, the bullwhip effect. Consequently, we develop a set of propositions to analyse and assess how fake news, misinformation and disinformation can disrupt the supply chain and propose appropriate risk management strategies (Fig. 2).

### Proposition 1

*Information is essential in all parts of the supply chain.*

While information and information risks have not been studied much, they are essential for all parts and processes of the supply chain. Decision-makers should manage information across all supply chain stages (Prakash et al., 2017). Spekman and Davis (2004) argued that information should be transparent and visible throughout the supply chain. Galbraith (1974) emphasises that information must be processed during tasks in the Information Processing Model. All changes in resource allocation, schedules, and priorities require information processing during task performance. In addition, there is a need for information processing to coordinate all interdependent tasks. Earlier articles in supply chain management (i.e. Kraljic, 1983) stressed the importance of considering the risks from interconnected flows of material, information and funds in networks, and this literature had gained momentum with many studies reporting disrupted supply chains and concepts for risk management strategies (Wagner & Bode, 2009).

**Fig. 3:**
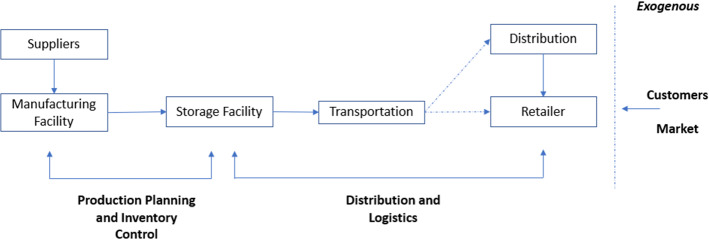
The supply chain framework

It is useful to highlight in practice the essential role of information in the supply chain, as depicted in Fig. 3. This slightly modified version of Beamon (1998) describes the supply chain processes. We include the customers who express the exogenous information from the market to the supply chain. The customers signal and provide information about their preferences mainly to the retailer. Then such information is transmitted endogenously to the supply chain, from retailers to transportation, storage, manufacturing and suppliers. Information is used for transportation, storage, manufacturing and ordering operations and processes. According to consumers’ demands, information might change and be processed accordingly. It is a simple example to highlight information’s role in every stage of the supply chain. However, the transmission channels and complexity of operation can be much greater in reality.

### Proposition 2

*Better quality information can reduce risks and disruptions in every part of the supply chain.*

**Fig. 4:**
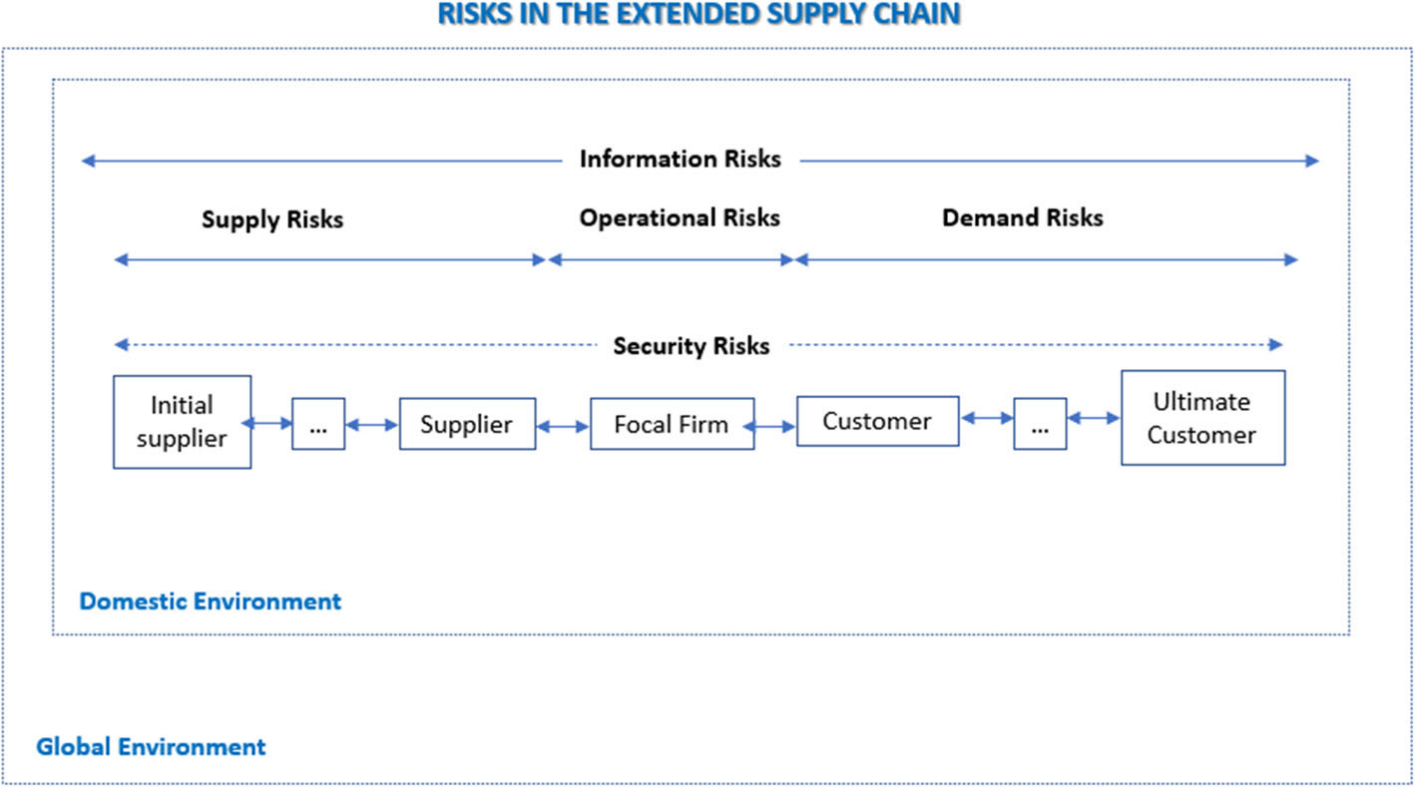
Information risks in the extended supply chain

Following the above, we argue that fake news, misinformation, and disinformation can disrupt every part of the supply chain. The severity of disruption depends on the quality of information. The literature focuses on specific aspects and risks, notably demand and supply risks (Shekarian & Mellat Parast, 2020; Tang & Nurmaya Musa, 2011). Nevertheless, information is critical to every node of decision-making, operation and process. In order to capture those risks and disruptions, we use a modified version of Manuj and Mentzer (2008) and Menzter (2001), described in Fig. 4. Most of these risks are overlapping and interdependent and do not exist in isolation (Manuj & Mentzer, 2008). For example, security risk includes Information systems and infrastructure security that largely overlap with information risks. Another advantage of this model is that it displays the domestic and global environment except for the extended supply chain. It is crucial for our analysis since it can analyse the exogenous risks and disruption resulting from low-quality information.

Supply, demand, operations, and security, and we argue that information risks are associated with supply chains and disrupt the operations of mainly matching supply with demand (Manuj & Mentzer, 2008). We also argue that information is an overarching theme and can largely influence other types of risks and their aspects. Demand and supply risks are concerned with potential disturbances in the flow of products and information (Shekarian & Mellat Parast, 2020). We further suggest that better quality information can avoid disturbances in the flow of products. Supply risks involve ‘the potential difference between actual and forecasted demand’ (Shekarian & Mellat Parast, 2020). More and better information can assist in forecasting and managing the ‘unanticipated or volatile demand, insufficient or distorted information from customers about orders or demand quantities’ (Shekarian and Parast, 2019) aspects of supply risks. In addition, supply risks and disruption of supplies in terms of time, quality, and quantity can be more effectively managed with better quality information. Finally, risks as a process encompassing aspects of the ‘reliability of the supporting communication system’ (Shekarian & Parast, 2019) can be reduced by having better information.

### Proposition 3a

*There is a higher risk, and the impact of disruptions on supply chains is more negative when those disruptions are due to exogenous fake news, misinformation and disinformation.*

### Proposition 3b

*There is a higher risk and the impact more negative of disruptions on supply chains when those disruptions are due to intentional rather than inadvertent wrong and misleading information.*

Propositions 3a and 3b are largely based on DuHadway et al. (2019) arguing that ‘The impact of disruptions on firm performance is more negative when those disruptions are due to an intentional rather than an inadvertent cause.’ We expand the scope of the proposition from the firm to the extended supply chain. The firm is included as part of the supply chain, and there can be different firms and organisations in the supply chain. Again, this depends on the scale and complexity of operations and supply chains. Nevertheless, the definition of an exogenous disruption occurs outside the supply chain, while an endogenous disruption occurs within the supply chain rather than the firm (DuHadway et al., 2019).

We also follow some methods DuHadway et al. (2019) proposed on different operational classifications of disruption events and risks. They are (i) Force Majeure disruption due to non-deliberate events outside of the supply chain, from accidental and exogenous causes and might include disruptions such as natural disasters, economic and political instability, port closures, terrorism, and are the most well-studied disruption within the literature. (ii) Performance Failure disruption is due to a negligent act that is endogenous to the supply chain. (iii) Targeted Strike disruption is caused by intentional and exogenous supply chain factors, such as competitors, government intervention cyberattacks, and industrial theft. (iv) Inside Jobs are intentional and endogenous to the supply chain, such as fraudulent behaviour between organisations (DuHadway et al. 2019).

Fake news and misinformation can have negative effects and disrupt supply chains. Nevertheless, we argue that intentional and exogenous disinformation can cause significant damage and substantial disruptions. It is because it corresponds to targeted strike disruption and inside jobs. An illustrative example is the Takata airbag scandal. The Takata airbags define a new level of deception, with effects increasing mortality risks to consumers and social impacts going beyond a global societal level (Hammadi et al., 2018). Takata is a notable case of intentionally wrong information within the extended supply chain. Takata was a renowned company producing airbags. Around 2000 the company noticed internally that airbag inflators were not functioning properly, and some erupted in tests, while in 2004, a Takata executive admitted to ‘manipulating’ airbag information (Jones & Bomey, 2022). Despite this information, Takata did not properly provide it to other partners n the supply chain and consumers. “Approximately 67 million Takata airbags (priority groups 1–12) have been recalled because these airbags can explode when deployed, causing serious injury or even death” and has affected many car manufacturers and models. (NHTSA, 2022).

Raman et al. (2020) use Greater London as a case study and show that disinformation can lead to energy disruptions and propagate in social networks, potentially amplifying the attack’s impact. Such energy shocks can be exogenous to supply chains and cause large disruptions. Similarly, Waniek et al. (2021) found that traffic networks are vulnerable to disinformation attacks. Consequently, this is another exogenous risk that can impact supply chains since transportation, as shown in the Fig. 3, is an important part. Finally, we would like to emphasise that disinformation can be highly sophisticated and complicated by combining different events and risks as targeted strike disruption, including an inside job. The risks and complexity of disinformation, deception and modes of cyber attacks have been described in other studies as Kello (2013), who mention supply chain risks and vulnerabilities due to information systems and offshore manufacturers and additional forms of attacks using information (i.e. Line X) and the disruptive potential of counterintelligence on material supply chains (Gartzke & Lindsay, 2015).

### Proposition 4

*Fake news, misinformation and disinformation can be amplified and cause larger supply chain disruptions.*

One of the key characteristics of fake news, misinformation and disinformation is that social media have created innovative communication channels and have contributed to the quick spread and amplification of low-quality information. Beyond the purpose of this paper and an area that requires future research, it is not easy to model and analyse in detail how low-quality information is transmitted within the extended supply chain. As discussed above, disinformation campaigns can affect various parts of the supply chain. In some cases, systemic effects can impact multiple parts and have a persistent character.

Nevertheless, what is important is that fake news, misinformation and disinformation can be distorted and amplified. We can distinguish different effects. One of the effects is that low-quality information can be further distorted in the supply chain. It might be due to a lack of understanding, problems with interpretability and a lack of effective information systems presented in the literature review. These distortion effects can be even stronger when fake news, misinformation and disinformation are further amplified by exogenous actors at different parts of the extended supply chain. In addition, amplification can come from within the supply chain.

Forrester (1961) argued that information, and for that reason, low-quality information, can be amplified. In addition, although he recognised that there could be external perturbations, he argued that not perfect and inaccurate (i.e. low-quality information) can be distorted, as modified and contain errors, but implied that this is mainly within the extended supply chain. Consequently, such low-quality information could create bullwhip effects and increase risks, further disrupting the supply chain. While Forrester mainly analyses the endogenous effects, we expand on the exogenous events. Finally, it should be argued that since information integrates other flows (Forrester, 1961), as material and financial, the amplification effects and disruption can be spread to other flows and operations and create even bigger disruptions.

### Proposition 5

*Supply Chain Visibility and Information sharing can be effective risk management strategies for all types of disruptions.*

SCRM has been defined “as management of risks through coordination and cooperation” (Tang, 2006). Therefore coordination and cooperation through sharing information can be an effective risk management strategy. Nevertheless, this assumes that they share good-quality information. If they share fake news, misinformation and disinformation, this can cause contagion effects, disruption in other parts of the supply chain, and amplification. Thus, sharing information is particularly useful when it concerns awareness and prevention of low-quality information. Moreover, it should be ensured that the shared information is of good quality within the supply chain, and in this direction, as we analyse below, Blockchain plays a protagonist role.

Another important aspect is to have good quality information about supply chain risks. Information about risks should include situational awareness and estimation of such risks. Fake news, misinformation, disinformation, and deception can undermine supply chain risks. In many cases, supply chain risks and disruptions might be attributed to suppliers or other partners in the supply chain rather than the firm itself. Therefore, good quality information about these risks is essential for detection.

Moreover, besides accurate information, timely information is valuable. It is especially true when risk is realised, and a supply chain disruption occurs. Quality information about the disruption is necessary for risk mitigation and recovery strategies. Finally, information sharing for IT systems is crucial. IT systems can improve visibility and effective information sharing, but they communicate information. Lack of availability or integrity of information can lead to disruptions in the supply chain. The literature relates related information risks to IT systems (Bugert & Rainer, 2018; Rajagopal et al., 2017; Tang & Nurmaya Musa, 2011). Sharing information about IT vulnerabilities and cybersecurity risks is necessary across the supply chain. It should be noted that cyber attackers use misinformation and, in particular, disinformation to infiltrate IT systems (Petratos, 2021). Blockchain can significantly reduce risks to IT systems and supply chains.

### Proposition 6

*Blockchain applications can manage information risks and reduce supply chain disruptions.*

Against all the information above supply chain risks, Blockchain can offer various solutions and improve risk management. Distributed Ledger Technologies (DLT), as Blockchain is known, allow visibility and ensure transparency while sharing information securely within a network. Firstly we would like to present some existing applications in operations management. Then we discuss and propose blockchain recommendations and strategies to improve risk management and resilience of supply chains. It should be emphasised that Blockchain is a rather new technology, especially concerning the applications to the supply chain.

With its relative advantages in terms of quality and transparency, blockchain technologies in manufacturing have become an excellent opportunity to increase the availability and quality of information for various processes. Some of these processes concern the monitoring and management of the supply chain and all its phases, from raw material to finished product (Litke et al., 2019); digital tracking of each movement of the goods to allow authorised personnel to access any information relating to the shipment, obtaining a guarantee of truthfulness (Tijan et al., 2019);. There is a range of additional tracking applications using Blockchain. Lucena et al. (2018) propose tracking, which has instant information on complete products from its electronic manufacturing service providers, and the Blockchain helps to track and authenticate them in real time.

Tracking asset maintenance can also be improved if an asset is mainly maintained according to the schedule, and notably, it supports multiple parties (Pundir et al., 2019). There is also tracking critical parameters (Kuhn et al., 2018) for a product sensitive, such as storage conditions. In combination with other emerging technologies, the Blockchain can help (i.e. IoT, AI, etc.) companies monitor conditions. Monitoring resource conditions using Blockchain and IoT helps monitor the conditions of assets in remote locations. (Alcarria et al., 2018). Such applications can be particularly useful for situational awareness and detecting supply chain risks. Similarly, the tracing of products is enabled by Blockchain, which allows the tracing of products or components by recording the entire production path of a product, from the origin of its components until the product reaches the consumer (Duan et al., 2020; Subramanian et al., 2020). Tracing of origin traces the attributes of a product and any change in ownership (Lu & Xu, 2017).

Another key area of applications is retail trade which focuses on digital markets and counterfeiting prevention. Counterfeiting is based on wrong and misleading information. Blockchain applications in this field include blockchain-enabled markets trust and; the prevention of counterfeit products (Kumar & Tripathi, 2019). With the Blockchain’s ability to trace the origin of each part of a final product, it is possible to have total control and visibility for all interested parties. Blockchain guarantees the authenticity of the goods and reduces counterfeiting and Inventory and theft tracking and tracking of returned goods (Chang et al., 2020). Blockchain also allows organisations to extend warranties to customers with genuine products and avoid losses in warranty fraud (Banerjee, 2018). Finally, it can keep the entire history of a product and allows not only the firm and consumers, as in previous cases, but also regulators to determine if that product has been manufactured and managed in a compliant manner (Gozman et al., 2020).

The ‘digital wire’ provides integrated visibility of assets (Satapathy et al., 2019). In contrast to management, Blockchain applications achieve decision efficiency, improve execution speed, and support faster dispute resolution (Guo et al., 2021). All these blockchain applications improve the security and integrity of information within parts of the extended supply chain. At the same time, some of these applications reduce the risks with exogenous factors and provide good quality information to customers and the market. Nevertheless, challenges remain. While the Blockchain can secure the information and communicate it effectively, this information should not be interfered with from the source.

Fake news, misinformation and disinformation should be avoided before reaching the Blockchain. Petratos (2021) proposes risk management strategies as he uses and develops anti-misinformation, disinformation, and fake news tools and technologies (i.e. bot/spam detection, credibility scoring, etc.) that can detect and track and prevent low-quality information. Good quality information can also be secured with Blockchain before reaching the supply chain. More innovation is required towards this area and should be encouraged by governments and other stakeholders. In addition, more investment is necessary for two reasons; first, because the risk of fake news, misinformation and disinformation has significantly grown and second, because some of the risk management and mitigation strategies as the Blockchain have not been developed yet. Protection against supply chain risks requires government and industry coordination, but such efforts have barely commenced and developed (Kello, 2013). Therefore, further sharing information, creating partnerships, and sufficiently investing to realise them can be valuable. (Petratos, 2021). However, this partnership and capacity development should be within the supply chain and beyond it, in the industry, and with government agencies, like the police and the military, to increase the resilience of supply chains (Table 1).

**Table 1:** Propositions and Relevant Literature Overview

No.	Propositions	Summary	Relevant literature
1	*Information is essential in all parts of the supply chain*	Information processing is an essential part of the supply chain. Therefore, preserving its integrity is an extremely critical task	Prakash et al. (2017), Spekman and Davis (2004), Galbraith (1974), Kraljic (1983), Wagner and Bode (2009), Beamon (1998)
2	*Better quality information can reduce risk and disruptions in every part of the supply chain*	Transmission mechanisms in the supply chain potentially allow bad information to disrupt the entire chain	Shekarian & Mellat Parast, 2020), Tang and Nurmaya Musa (2011), Manuj and Mentzer (2008), Menzter (2001)
3a	*There is a higher risk, and the impact of disruptions on supply chains is more negative when those disruptions are due to exogenous fake news, misinformation and disinformation*	Exogenous and intentional mislaedng information prove more dangerous than endogenous and inadvertent ones	DuHadway et al. (2019), Hammadi et al., (2018), Jones and Bomey (2022), NHTSA (2022), Waniek et al. (2021), Kello (2013), Gartzke and Lindsay (2015)
3b	*There is a higher risk and the impact more negative of disruptions on supply chains when those disruptions are due to intentional rather than inadvertent wrong and misleading information*		
4	*Fake news, misinformation and disinformation can be amplified and cause larger supply chain disruptions*	Media and Social Media are the most affected industries and play a pivotal role in amplifying the effect of fake news, misinformation and disinformation	Forrester (1961), Gradoń et al. (2021), Landon-Murray et al. (2019), Shu et al. (2020)
5	*Supply Chain Visibility and Information Sharing can be effective risk management strategies for all types of disruptions*	Information sharing for IT systems is crucial. Sharing information about IT vulnerabilities and cybersecurity risks is necessary across the supply chain. It should be noted that cyber attackers use misinformation and, in particular, disinformation to infiltrate IT systems	Tang (2006), Bugert and Rainer (2018), Rajagopal et al. (2017), Tang and Nurmaya Musa (2011), Petratos (2021)
6	*Blockchain applications can manage information risks and reduce supply chain disruptions*	Blockchain can significantly reduce risks to IT systems and supply chains. Distributed Ledger Technologies (DLT), as Blockchain is known, allow visibility and ensure transparency while sharing information securely within a network Among the applications: monitoring and management of all supply chain phases, obtaining a guarantee of truthfulness, tracking asset maintenance, tracking critical parameters, monitoring resource conditions, tracing of products, and prevention of counterfeit products. It guarantees goods’ authenticity and reduces counterfeiting and Inventory and theft tracking and tracking of returned goods. It can keep the entire history of a product and allows the firm and consumers, as in previous cases, and regulators to determine if that product has been manufactured and managed in a compliant manner	Litke et al. (2019), Tijan et al. (2019), Lucena et al. (2018), Pundir et al. (2019), Kuhn et al. (2018), Alcarria et al. (2018), Duan et al. (2020), Subramanian et al. (2020), Lu and Xu (2017), Kumar and Tripathi (2019), Chang et al. (2020), Banerjee (2018), Gozman et al. (2020), Satapathy et al. (2019), Guo et al. (2021), Petratos (2021), Kello (2013)

## Conclusion, limitations and future research

Fake news, misinformation and disinformation have become significant problems for societies and supply chains. Moreover, their risks are increasing. It is highlighted by the ‘infodemic’ of COVID-19 but also in the news and literature. This paper examines the disruption and risks to supply chains and makes three distinct contributions. First, we review the literature on information and supply chains and find that information flows and risks are attracting less attention. We contribute to this literature, mainly SCRM and SCRES, by further analysing it and suggesting that information integrates other flows, processes and operations, and it is an overarching theme that is essential in every part of the supply chain. In addition, previous research on risks lacks a cohesive theoretical framework (DuHadway et al. 2019) and is based on related studies. We expand on creating a theoretical framework incorporating fake news, misinformation and disinformation.

The second main contribution is analysing low-quality information and its risks and disruption on the supply chain. We examine fake news, misinformation and disinformation, their characteristics, risk and disruptions to supply chains. We find that there is a higher risk, and the impact of disruptions on supply chains is more negative when those disruptions are due to exogenous fake news, misinformation and disinformation and when those disruptions are intentional. Fake news, misinformation and disinformation can be amplified and cause larger supply chain disruptions. These findings answer the research question: What are the information risks, and how can they disrupt supply chains?

The third contribution comes from exploring the question of how blockchain technologies improve information management risk management and increase supply chain resilience. The framework presented in this paper provides a useful tool for managing risks due to misleading information disruptions in practice. We present some applications of Blockchain to the supply chain and find support that Blockchain can advance the risk management and resilience of supply chains. Moreover blockchain can have practical benefits by facilitating cooperation and partnerships among different supply chain participants. Another practical implication for decision makers is that information sharing is an effective strategy for supply chain risk management. Nevertheless, this study comes with two main limitations. It is a theoretical study, and blockchain applications to supply chains are a novice and arguably not very mature. Future research can provide empirical evidence and expand supply chains’ different models and applications. Moreover, future research can expand and combine blockchain technology with other Industry 4.0 technologies for more effective SCRM.
